# Recent advances in the management of renal cell carcinoma

**DOI:** 10.12688/f1000research.7935.1

**Published:** 2016-03-23

**Authors:** Ana M. Molina, David M. Nanus

**Affiliations:** 1 Division of Hematology and Medical Oncology, Department of Medicine, Weill Cornell Medicine, 1300 York Avenue, New York, NY, 10065 , USA; 2 Meyer Cancer Center, Weill Cornell Medicine, 1300 York Avenue, New York, NY, 10065 , USA

**Keywords:** renal cell carcinoma, cancer, kidney, therapy, management

## Abstract

Therapeutic options for patients with metastatic renal cell carcinoma have significantly improved over the past few years with the recent approval of two new agents resulting in prolonged progression-free and overall survival.

## Introduction

In 2016, there will be an estimated 62,700 new cases of kidney cancer and over 14,000 deaths ^1^. Clear cell renal cell carcinoma (ccRCC) is the most common cancer of the kidney. The mainstay of treatment for many years was cytokine therapy with interferon alpha (IFN-α) and interleukin-2 (IL-2). Before the year 2000, high-dose IL-2 was the only approved treatment for patients with metastatic RCC (mRCC) based on objective response rates (ORRs) of 10% and 15% with complete and durable responses reported ^2– 4^. However, identification of the Von Hippel-Lindau (VHL) tumor-suppressor gene, and that its inactivation in ccRCC led to increased expression of hypoxia-inducible factor alpha (HIF-α) and angiogenesis-related proteins such as vascular endothelial growth factor (VEGF) and platelet-derived growth factor B chain (PDGF-B), led to the development of targeted therapies that specifically inhibit VEGF signaling pathways. Today, most patients with mRCC are treated with sunitinib, pazopanib, or bevacizumab as first-line therapy based on phase III randomized studies that have demonstrated significant improvement in progression-free survival (PFS) and/or overall survival ^5^. Until recently, patients who progressed on first-line therapy subsequently received the mTOR inhibitor everolimus based on a 2008 randomized phase III study that demonstrated a median PFS of 4.9 months in patients receiving everolimus versus 1.9 months in patients on placebo ^6^. A number of recent studies have changed this paradigm and expanded the therapeutic option for patients who have progressed on first-line anti-VEGF therapy. This review will summarize these data.

## First-line therapy for mRCC

Sunitinib was approved by the US Food and Drug Administration (FDA) in 2006 for the treatment of patients with mRCC and became a standard first-line therapy. Pazopanib, another multi-kinase inhibitor targeting VEGF receptor (VEGFR), PDGF receptor (PDGFR), and c-KIT, was approved by the FDA for the treatment of advanced RCC in 2009. The COMPARZ phase III study compared the efficacy and safety of pazopanib and sunitinib as first-line therapy ^7^. In this trial, ORRs were 31% for pazopanib and 24% for sunitinib. Pazopanib was non-inferior to sunitinib with a median PFS of 8.4 months and 9.5 months, respectively. Overall survival was similar in the two groups.

Bevacizumab, a humanized VEGF-neutralizing antibody, was FDA approved in 2009 based on two multicenter phase III studies comparing bevacizumab plus IFN to IFN alone as first-line treatment in patients with mRCC ^8, 9^. Both studies demonstrated a significant improvement in PFS in patients receiving bevacizumab (10.2 versus 5.4 months and 8.5 versus 5.6 months) as well as an increase in the objective tumor response rate (30.6% versus 12.4% and 25.5% versus 13.1%). Based on these trials, sunitinib, pazopanib, and bevacizumab plus IFN are each considered an option for first-line therapy in patients with mRCC ^5^.

## Second-line therapy after anti-VEGF therapy for mRCC

Until 2012, everolimus was the only second-line therapy to demonstrate improvement in PFS after first-line anti-VEGF therapy. Axitinib, another VEGFR kinase inhibitor, was approved in 2012 for the treatment of mRCC following failure of a prior systemic therapy based on results from the Axitinib Versus Sorafenib (AXIS) trial, a global, randomized phase III trial comparing axitinib with sorafenib as second-line therapy in patients with treatment-refractory mRCC ^10^. Median PFS was significantly longer in patients treated with axitinib versus sorafenib (6.7 versus 4.7 months). Importantly, this PFS benefit was significant in patients who had previously received treatment with cytokines (12.1 versus 6.5 months) or sunitinib (4.8 versus 3.4 months). Axitinib also led to a significantly higher ORR.

## Emerging new agents

Although VEGF-targeted agents have significantly impacted patients with mRCC, most patients fail to achieve a complete response, long-term survival rates remain low, and most patients develop resistance. Consequently, the search for newer agents has continued. Cabozantinib is a small-molecule tyrosine kinase inhibitor (TKI) that targets VEGFR, as well as MET and AXL, each of which has been implicated in the development of resistance to anti-angiogenic drugs ^11^. Cabozantinib first demonstrated anti-tumor activity in heavily pretreated RCC patients with a response rate of 28% and median PFS of 12.9 months ^12^. A recent randomized phase III trial (METEOR) compared the efficacy of cabozantinib with that of everolimus in patients with RCC who had progressed after VEGFR-targeted therapy. In this trial, patients treated with cabozantinib demonstrated 21% ORR and a median PFS of 7.4 months, while patients treated with everolimus experienced a 5% ORR and a median PFS of 3.8 months ^11^. PFS benefit was consistent in subgroup analyses independent of Memorial Sloan-Kettering Cancer Center (MSKCC) risk group and Eastern Cooperative Oncology Group (ECOG) status, organ involvement including bone and tumor burden, and extent of prior VEGFR-TKI and prior programmed cell death protein 1 (PD-1)/PD-L1 therapy ^13^. The overall survival data at the time of the pre-specified interim analysis were immature. However, there was a strong trend toward longer survival in patients treated with cabozantinib. Common adverse events with cabozantinib included fatigue, diarrhea, nausea, decreased appetite, hypertension, and hand-foot syndrome ^11^. Dose reductions occurred in 60% of patients who received cabozantinib and in 10% of those treated with everolimus.

The clinical development of immune checkpoint inhibitors has led investigators to revisit the role of immunotherapy in RCC. Nivolumab is a human monoclonal antibody that targets the co-inhibitory receptor PD-1, which is expressed on activated T cells ^14^. Upregulation of PD-1 expression in tumor lymphocytes is associated with aggressive disease and poor prognosis in RCC ^15^. Nivolumab first demonstrated anti-tumor activity and durable responses in 9 out of 33 patients (27%) with RCC ^16^. Nivolumab resulted in objective responses in 20 to 22% of patients with mRCC and overall survival ranging from 18.2 to 25.5 months in a phase II dose-ranging trial ^17^. Recently, a randomized phase III trial (CheckMate 025) compared nivolumab with everolimus in patients with RCC previously treated with one or two anti-angiogenic regimens ^18^. In this trial, patients treated with nivolumab demonstrated a 25% ORR, median PFS of 4.6 months, and overall survival of 25 months, while patients treated with everolimus experienced a 5% ORR, a median PFS of 4.4 months, and overall survival of 19.6 months. Consistent with the benefit observed in the overall population of CheckMate 025, nivolumab demonstrated both an overall survival and an ORR benefit across key subgroups including risk groups, number and sites of metastases, and prior therapies ^19^. Fatigue, nausea, and pruritus were the most common treatment-related adverse events in patients treated with nivolumab. Eight percent of patients discontinued treatment with nivolumab owing to treatment-related adverse events. Based on the positive results, the trial was stopped early and nivolumab was granted breakthrough therapy designation from the FDA for advanced RCC in 2015.

## The changing paradigm for mRCC treatment

The introduction of targeted therapies for the treatment of mRCC has vastly changed the treatment landscape of this disease. Now, with the availability of seven approved targeted agents and two approved immunotherapy agents, clinicians must consider the best way to incorporate these therapies into the management of patients with mRCC. Clinicians are now faced with questions such as how many therapies can a patient receive and what is the optimal sequence of treatment? Results from recent phase III clinical trials have established the role of targeted agents in the management of advanced RCC in the first- and second-line settings. The survival benefit and favorable safety profile demonstrated in the CheckMate 025 phase III trial supports nivolumab as a new standard of care for patients with advanced RCC in the second-line setting. The response and PFS data on cabozantinib are striking. The survival data for cabozantinib, when mature and if positive, will provide a new treatment option for second-line therapy as well. In the short term, patient preference (oral versus intravenous administration) and cost will play a role in treatment decision making. Ongoing studies are investigating optimal sequential therapy and combination therapy with existing and novel targeted and immunotherapy agents. In addition, studies identifying prognostic factors, biomarkers, and mechanisms of resistance are underway.

## Abbreviations

ccRCC – Clear Cell Renal Cell Carcinoma

IFN – Interferon

IL – Interleukin

mRCC **–** Metastatic Renal Cell Carcinoma

ORR – Objective Response Rate

PDGFR – Platelet-Derived Growth Factor Receptor

PD-1 – Programmed cell death protein 1

PFS – Progression-Free Survival

RCC – Renal Cell Carcinoma

TKI – Tyrosine Kinase Inhibitor

VEGF – Vascular Endothelial Growth Factor

VEGFR – Vascular Endothelial Growth Factor Receptor
